# Non-AIDS defining cancers in the D:A:D Study - time trends and predictors of survival: a cohort study

**DOI:** 10.1186/1471-2334-13-471

**Published:** 2013-10-09

**Authors:** Signe W Worm, Mark Bower, Peter Reiss, Fabrice Bonnet, Matthew Law, Gerd Fätkenheuer, Antonella d’Arminio Monforte, Donald I Abrams, Andrew Grulich, Eric Fontas, Ole Kirk, Hansjakob Furrer, Stephane De Wit, Andrew Phillips, Jens D Lundgren, Caroline A Sabin

**Affiliations:** 1 Copenhagen HIV Programme, University of Copenhagen, Copenhagen, Denmark; 2 Chelsea and Westminster Hospital, London, UK; 3 Academic Medical Center, Amsterdam, The Netherlands; 4 ISPED, Universite Victor Segalen, Bordeaux, France; 5 Kirby Institute, University of New South Wales, Sydney, Australia; 6 University Hospital of Cologne, Cologne, Germany; 7 Hospital San Paolo, University of Milan, Milan, Italy; 8 Hematology-Oncology, San Francisco General Hospital/University of California, San Francisco, USA; 9 CHU Nice Hopital de l’Archet, Nice, France; 10 Department of Infectious Diseases, Bern University Hospital and University of Bern, Bern, Switzerland; 11 Department of Infectious Diseases, CHU Saint-Pierre Hospital, Bruxelles, Belgium; 12 Research Department of Infection and Population Health, University College London, Royal Free Campus, Rowland Hill Street, London NW3 2PF, UK

**Keywords:** HIV, Non-AIDS defining cancers, Incidence, Trends, Prognosis

## Abstract

**Background:**

Non-AIDS defining cancers (NADC) are an important cause of morbidity and mortality in HIV-positive individuals. Using data from a large international cohort of HIV-positive individuals, we described the incidence of NADC from 2004–2010, and described subsequent mortality and predictors of these.

**Methods:**

Individuals were followed from 1st January 2004/enrolment in study, until the earliest of a new NADC, 1st February 2010, death or six months after the patient’s last visit. Incidence rates were estimated for each year of follow-up, overall and stratified by gender, age and mode of HIV acquisition. Cumulative risk of mortality following NADC diagnosis was summarised using Kaplan-Meier methods, with follow-up for these analyses from the date of NADC diagnosis until the patient’s death, 1st February 2010 or 6 months after the patient’s last visit. Factors associated with mortality following NADC diagnosis were identified using multivariable Cox proportional hazards regression.

**Results:**

Over 176,775 person-years (PY), 880 (2.1%) patients developed a new NADC (incidence: 4.98/1000PY [95% confidence interval 4.65, 5.31]). Over a third of these patients (327, 37.2%) had died by 1st February 2010. Time trends for lung cancer, anal cancer and Hodgkin’s lymphoma were broadly consistent. Kaplan-Meier cumulative mortality estimates at 1, 3 and 5 years after NADC diagnosis were 28.2% [95% CI 25.1-31.2], 42.0% [38.2-45.8] and 47.3% [42.4-52.2], respectively. Significant predictors of poorer survival after diagnosis of NADC were lung cancer (compared to other cancer types), male gender, non-white ethnicity, and smoking status. Later year of diagnosis and higher CD4 count at NADC diagnosis were associated with improved survival. The incidence of NADC remained stable over the period 2004–2010 in this large observational cohort.

**Conclusions:**

The prognosis after diagnosis of NADC, in particular lung cancer and disseminated cancer, is poor but has improved somewhat over time. Modifiable risk factors, such as smoking and low CD4 counts, were associated with mortality following a diagnosis of NADC.

## Background

Since the introduction of combination antiretroviral therapy (cART), there has been a dramatic decrease in the incidence of AIDS-related morbidity and mortality in HIV-positive patients [1]. However, while the incidence of AIDS-defining cancers (ADC) has steadily declined, there have been reports of an apparent increase in the number of non-AIDS-defining cancers (NADC) [2-11] and increases in cancer-related mortality [12,13]. This has led to new challenges for those responsible for the management and treatment of HIV-positive individuals.

There are several factors to consider in assessing trends in incidence of NADC. As age is an important risk factor for the development of many NADC, and as the proportion of HIV-positive patients above the age of 50 has increased, any apparent increase in incidence may in part simply reflect the normal ageing process in this population [4,14]. Furthermore, given the lifestyle characteristics of many individuals living with HIV, the increase in survival due to cART may also result in increased exposure of the population to oncogens such as viral co-infections, tobacco, alcohol and sun exposure, which may all contribute to an increased incidence of NADC [15,16].

In the pre-/early-cART era, the survival of HIV-positive patients diagnosed with cancer was significantly poorer than that of uninfected patients with cancer [17,18]. These findings may be attributable to more advanced cancer stage and low performance status at cancer diagnosis and other HIV-associated opportunistic diseases among HIV-positive patients [17,19] as well as the impact of an increased burden of traditional risk factors, such as smoking [14,20], in this population. However, there are only limited data on the underlying causes of death in patients with a NADC in the setting of HIV.

We studied a large cohort of HIV-positive individuals across Europe and Australia to assess the incidence of NADC over the period 2004–2010 and to describe mortality rates, and predictors of mortality, after a diagnosis of cancer.

## Methods

The D:A:D Study is a prospective, observational study formed by the collaboration of 11 cohorts that follow HIV-positive persons from 212 clinics in Europe, Australia and the United States. The main objective of the D:A:D Study is to assess the incidence of myocardial infarction and other cardiovascular disease endpoints in HIV-positive persons, and to describe associations between these endpoints and antiretroviral therapy [21]. However, following reports of a possible relationship between the use of antiretroviral therapy and other long-term adverse effects, the study protocol was modified in September 2008 to include the collection of information on three additional endpoints: NADC, end-stage renal disease and chronic liver disease. The primary objective of the analyses of the NADC endpoint is to assess the possible relationship between exposure to cART and the risk of NADC, with a secondary objective being to assess the possible association between immunodeficiency and NADC. These analyses are ongoing and will be reported separately. All participating cohorts followed local national guidelines/regulations regarding patient consent and/or ethical review. In particular, of the countries represented by the participating cohorts, only Switzerland and Australia require specific ethical approval for D:A:D in addition to that required for their national cohorts (Swiss HIV Cohort Study and AHOD), both of which have obtained this approval. France, Italy, and Belgium do not require specific ethical approval over-and-above that required for the individual cohorts (Nice/Aquitaine, Brussels St. Pierre and IcoNA, respectively). Neither the Netherlands (ATHENA) nor the United States (CPCRA) require specific ethical approval as data are provided as part of HIV care in the first instance, and as the datasets are non-identifiable public use datasets in the second instance. For the EuroSIDA study (which includes the data from the BASS and Swedish cohorts), which contains participants from across many European countries, each participating site has a contractual obligation to ensure that data collection and sharing is done in accordance with national legislation; each site principal investigator either maintains appropriate documentation from an ethical committee (if required by law) or has a documented written statement to say that this is not required.

### End-points

Eight of the 11 D:A:D cohorts have provided ongoing information on all new NADC (any non-AIDS malignant disease other than basal or squamous cell skin cancer and pre-cancers) occurring after the protocol change on 1st February 2008 (the Swiss HIV Cohort, ATHENA, the Nice HIV Cohort, the ANRS C03 Aquitaine Cohort, EuroSIDA, the Australian HIV Observational Database, the ICONA Foundation Study and the Brussels St. Pierre Cohort). However, as the eight cohorts had been systematically collecting information on all new NADC that occurred from 2004 or earlier, information was also collected retrospectively on events occurring from 1st January 2004 until 31st January 2008.

Detailed information on all NADC is collected on a specific case report form (CRF); information collected includes date of diagnosis, type/location of cancer and histology/cytology report or other diagnostic method. Stage of disease (localised, disseminated or unknown) was available for some specific types of cancers only (particularly lung/anal cancers and Hodgkin’s lymphoma). Written guidelines for the completion of CRFs were distributed in 2007, and training was provided to staff at participating cohorts prior to data collection. Intensive quality assurance, including event monitoring, took place in 2009/2010. All reported events are evaluated and classified, where possible, as *definite*, *possible* or *probable* cancers by an independent committee that includes an oncologist. Classification reflects the degree of certainty in the NADC diagnosis:

*Definite:* based on supportive histology/cytology reports or a detailed summary of histopathological findings;

*Possible:* based on a precise clinical description of the case, and where treatment had been initiated to support the description of an invasive NADC, but lacking supportive histopathological findings;

*Probable:* based on clinical suspicion, biochemical or ultrasound findings.

The present analysis is based on data collected up to 1st February 2010. By that date, reports had been received on 1993 potential NADC; after excluding NADC events occurring prior to baseline (the latest of 1 January 2004 or entry in the D:A:D Study) and apparent NADC events that were subsequently rejected as they did not meet the study criteria for a definite, possible or probably NADC (AIDS-defining cancers, dysplasias, subsequent events following a first event, *in situ* cancers particularly for the anal and head/neck regions), 880 NADC were ultimately included in analyses. The majority of rejected events occurred prior to baseline. Of the 880 included events, 472 were accompanied by a completed CRF and could be classified as either definite (n = 383, 81.3%), probable (n = 38, 8.1%) or possible (n = 44, 9.3%); 1 event was a known relapse and 6 were unclassifiable even with the completed CRF. The remaining 408 events without a CRF were recorded as unclassifiable.

Information on cause of death was captured using The Coding Causes of Death in HIV (CoDe) form [22].

### Statistical methods

Follow-up for the analyses of NADC incidence started on the latest of 1st January 2004 or the date of enrolment in the D:A:D Study, and finished at the earliest of the date of diagnosis of a new NADC, 1st February 2010 (the cut-off date for the present dataset), death or six months after the patient’s last clinic visit. Crude incidence rates were estimated for each year of follow-up from 2004, overall and stratified by gender, age attained and mode of HIV acquisition. Incidence rates were also estimated separately for the three most frequently occurring cancers (Hodgkin’s lymphoma, anal and lung cancers). Age-adjusted incidence rates (adjusted to the age distribution of the cohort in 2004) were also estimated for all NADC combined. As it was not possible to estimate age-adjusted rates for specific cancers due to the small number of these events, the impact of the ageing population on calendar trends for specific cancers was investigated via Poisson regression with adjustment for age (as a time-updated covariate).

Cumulative risk of all-cause mortality following NADC diagnosis was summarised using Kaplan-Meier methods. Follow-up for these analyses started on the date of NADC diagnosis and finished at the earliest of the patient’s death, 1st February 2010 or 6 months after the patient’s last clinic visit. Factors associated with mortality following NADC diagnosis were identified using Cox proportional hazards regression, with the following factors at NADC diagnosis considered for inclusion: gender, mode of HIV acquisition, ethnic group (categorised as white/non-white), smoking status (current smoker, ex-smoker, never smoker, unknown), hepatitis C virus (HCV) coinfection status (positive: seropositive and HCV RNA positive or HCV RNA unknown; negative: seronegative, or seropositive but HCV RNA negative; or not tested) and hepatitis B virus (HBV) coinfection status (positive: active infection [HB surface antigen, HBe antigen, or HBV DNA positive]; positive: inactive infection [HB surface antigen negative, anti-HBe antibody positive, or HBV DNA negative]; negative or vaccinated; or not tested), age at diagnosis, year of diagnosis, the nadir and latest CD4 count and latest HIV RNA level at diagnosis, whether the patient had a prior ADC/NADC and the type of NADC. Note that cART status at NADC diagnosis was not included in these analyses as the majority of patients were receiving cART. Analyses of survival after a diagnosis of the specific cancers additionally considered stage of disease (disseminated, localised or unknown), specific type of cancer (for lung: adenocarcinoma, small cell or unknown; for Hodgkin’s lymphoma: nodular sclerosis, other or unknown) and, for Hodgkin’s lymphoma only, the haemoglobin level at diagnosis. Those factors that were associated with mortality in univariate analyses (p < 0.2) were considered for inclusion in a multivariable model. A backwards stepwise approach was then used to identify the factors independently associated with mortality following cancer diagnosis (p-value for retention in model = 0.1). The assumption of proportional hazards was tested through the inclusion of interaction terms between each covariate and the log of time in the main model. As sensitivity analyses, we also repeated our analyses to identify predictors of mortality from cancer only.

All analyses were performed using SAS version 9.3.

## Results

The eligible population (8 of the 11 D:A:D cohorts) includes 41,746 HIV-positive individuals (Table 1). At baseline, the average age of participants was 39 years (inter-quartile range (IQR) 33–46) and 73.2% were male. The median (IQR) CD4 count and HIV RNA at baseline were 434 (282–620) cells/mm^3^ and 2.3 (1.7-4.3) log_10_ copies/ml. Only 1.4% and 5.8% of participants, respectively, had a prior NADC or ADC.

**Table 1:** Baseline characteristics of study participants, overall and stratified according to whether they developed a NADC during prospective follow-up

		**All patients**	**Developed a NADC**	
			**Yes**	**No**
Total number		41746 (100.0)	880 (100.0)	40866 (100.0)
Gender*, n (%)	Male	30572 (73.2)	708 (80.5)	29864 (73.1)
	Female	11143 (26.7)	172 (19.6)	10971 (26.9)
Age (years)	Median (IQR)	39 (33, 46)	47 (41, 56)	39 (33, 46)
Mode of HIV acquisition, n (%)	Men having sex with men	18251 (43.7)	408 (46.4)	17843 (43.7)
	Injection drug user	6075 (14.6)	153 (17.4)	5922 (14.5)
	Heterosexual	14621 (35.0)	256 (29.1)	14365 (35.2)
	Other/unknown	2799 (6.7)	63 (7.2)	2736 (6.7)
Ethnic group, n (%)	White	20870 (50.0)	479 (54.4)	20391 (49.9)
	Black	2866 (6.9)	27 (3.1)	2839 (7.0)
	Other	838 (2.0)	11 (1.3)	827 (2.0)
	Unknown/not collected	17172 (41.1)	363 (41.3)	16809 (41.1)
BMI (kg/m^2^), n (%)	<18	1241 (3.0)	39 (4.4)	1202 (2.9)
	≥18, ≤26	24674 (59.1)	521 (59.2)	24153 (59.1)
	>26, ≤30	4751 (11.4)	99 (11.3)	4652 (11.4)
	>30	1643 (3.9)	43 (4.9)	1600 (3.9)
	Unknown	9437 (22.6)	178 (20.2)	9259 (22.7)
CD4 count (cells/mm^3^)	Median (IQR)	434 (282, 620)	393 (235, 583)	435 (284, 621)
Nadir CD4 count (cells/m^3^)	Median (IQR)	223 (97, 377)	144 (50, 290)	225 (99, 379)
HIV RNA (log_10 _copies/ml)	Median (IQR)	2.3 (1.7, 4.3)	1.7 (1.7, 3.9)	2.3 (1.7, 4.3)
Prior NADC, n (%)		585 (1.4)	48 (5.5)	537 (1.3)
Prior ADC, n (%)		2436 (5.8)	91 (10.3)	2345 (5.7)
Smoking status, n (%)	Current Smoker	15526 (37.2)	381 (43.3)	15145 (37.1)
	Ex-smoker	8121 (19.5)	226 (25.7)	7895 (19.3)
	Never smoked	10227 (24.5)	151 (17.2)	10076 (24.7)
	Unknown	7872 (18.9)	122 (13.9)	7750 (19.0)
HCV positive, n (%)		4431 (10.6)	105 (11.9)	4326 (10.6)
HBV positive, n (%)		1751 (4.2)	56 (6.4)	1695 (4.2)
Any use of ARV, n (%)		27778 (66.5)	718 (81.6)	27060 (66.2)

*Information on gender is missing for 31 participants.

The study participants were followed for 176,775 person-years (PY) (median [IQR] 5.0 [2.4-6.1]); the proportion of participants in whom follow-up was censored more than a year prior to death or the administrative censoring date for the cohort (assumed lost-to-follow-up) ranged from 1.9%-4.5% in each year. Over the study period, 880 (2.1%) participants developed a new NADC (incidence: 4.98/1000 PY, 95% confidence interval [CI] [4.65-5.31]). The three most frequent NADC were lung cancer (n = 140, 0.79 [0.66-0.92]/1000 PY), Hodgkin’s lymphoma (n = 112, 0.63 [0.52-0.75]/1000PY), and anal cancer (n = 79, 0.45 [0.35-0.55]/1000PY) (Table 2). Also shown in Table 2 is the number of participants that developed an ADC (n = 621) and type of ADC over follow-up. Characteristics of participants at NADC diagnosis, stratified by the type of NADC, are shown in Table 3. There were some notable differences: participants with Hodgkin’s lymphoma tended to be younger at diagnosis than those with other cancer types; those with anal cancer had lower median nadir CD4 cell count at NADC diagnosis; a higher percentage of participants with anal cancer had previously had an ADC. Information on stage of disease was available for 163 participants with lung/anal cancer and Hodgkin’s lymphoma; where known, the majority of lung cancers (76.3%) and Hodgkin’s lymphomas (83.3%) were disseminated, whereas the majority of anal cancers were localized (60.8%).

**Table 2:** Summary of NADC and ADC reported in the D:A:D study from 2004-2010

	**n (% of total events)**
*Number of NADC events*	*880 (100.0)*
Lung cancer	140 (15.9)
Hodgkin’s lymphoma	112 (12.7)
Anal cancer	79 (9.0)
Head and neck cancers	71 (8.1)
Liver cancer	59 (6.7)
Prostate	57 (6.5)
Breast cancer	43 (4.9)
Malignant melanoma	31 (3.5)
Colon cancer	26 (3.0)
Bladder cancer	21 (2.4)
Rectal	20 (2.3)
Gynecological cancers*	14 (1.6)
Stomach	13 (1.5)
Penile cancer	12 (1.4)
Kidney cancers	12 (1.4)
Acute myeloid leukemia	11 (1.3)
Metastasis of adenocarcinoma	9 (1.0)
Testicular	9 (1.0)
Lip cancer	6 (0.7)
Uterus	5 (0.6)
Brain cancer	5 (0.6)
Multiple myeloma	5 (0.6)
Metastasis of other cancer type	5 (0.6)
Metastasis of squamous cell carcinoma	5 (0.6)
Metastasis unspecified	4 (0.5)
Chronic lymphatic leukemia	2 (0.2)
Chronic myeloid leukemia	2 (0.2)
Connective tissue cancer	2 (0.2)
Bone cancer	1 (0.1)
Acute lymphatic leukemia	1 (0.1)
Leukemia unspecified	1 (0.1)
Type unknown	8 (0.9)
Unknown primary cancer	7 (0.8)
Other**	82 (9.3)
*Number of ADC events*	*621 (100.0)*
Kaposi’s sarcoma	331 (53.3)
Non-Hodgkin’s lymphoma	251 (40.4)
Cervical carcinoma	46 (7.4)

*Other than uterus and cervical cancer.

**Esophageal cancer, pancreas cancer, gall bladder cancer.

**Table 3:** Selected characteristics of study participants at the time of NADC diagnosis

		**Lung cancer**	**Hodgkin’s lymphoma**	**Anal cancer**	**Any NADC**
Total number		140 (100.0)	112 (100.0)	79 (100.0)	880 (100.0)
Gender, n (%)	Male	120 (85.7)	95 (84.8)	69 (87.3)	708 (80.5)
Female		20 (14.3)	17 (15.2)	10 (12.7)	173 (19.5)
Age (years)	Median (IQR)	54 (47, 59)	43 (38, 50)	48 (44, 51)	50 (44, 59)
Mode of HIV acquisition, n (%)	Men having sex with men	63 (45.0)	61 (54.5)	52 (65.8)	408 (46.4)
Injection drug user		25 (17.9)	15 (13.4)	15 (19.0)	153 (17.4)
Heterosexual		39 (27.9)	29 (25.9)	7 (8.9)	256 (29.1)
Other/unknown		13 (9.3)	7 (6.3)	5 (6.3)	63 (7.2)
Ethnic group, n (%)	White	72 (51.4)	70 (62.5)	46 (58.2)	479 (54.4)
Black		1 (0.7)	4 (3.6)	1 (1.3)	27 (3.1)
Other		0 (-)	6 (5.4)	0 (-)	11 (1.3)
Unknown/not collected		67 (47.9)	32 (28.6)	32 (40.5)	363 (41.3)
BMI (kg/m^2^), n (%)	<18	10 (7.1)	3 (2.7)	3 (3.8)	46 (5.2)
≥18, ≤26		64 (45.7)	69 (61.6)	48 (60.8)	439 (49.9)
>26, ≤30		9 (6.4)	17 (15.2)	5 (6.3)	79 (9.0)
>30		6 (4.3)	5 (4.5)	3 (3.8)	43 (4.9)
Unknown		51 (36.4)	25 (22.3)	20 (25.3)	273 (31.0)
CD4 count (cells/mm^3^)	Median (IQR)	365 (230, 487)	274 (167, 451)	380 (253, 580)	392 (245, 580)
Nadir CD4 count (cells/m^3^)	Median (IQR)	108 (53, 212)	134 (49, 232)	50 (16, 20)	127 (49, 245)
HIV RNA (log_10_ copies/ml)	Median (IQR)	1.7 (1.7, 2.3)	1.7 (1.7, 3.8)	1.7 (1.7, 2.0)	1.7 (1.7, 2.4)
Prior NADC, n (%)		8 (5.7)	4 (3.6)	4 (5.1)	48 (5.5)
Prior ADC, n (%)		9 (6.4)	11 (9.9)	15 (19.0)	91 (10.3)
Year of diagnosis, n (%)	2004-2005	46 (32.9)	31 (27.7)	20 (25.3)	238 (27.0)
2006-2007		45 (32.1)	54 (48.2)	33 (41.7)	339 (38.5)
2008-2010		49 (35.0)	27 (24.1)	26 (32.9)	303 (34.4)
Smoking status, n (%)	Current Smoker	71 (50.7)	45 (40.2)	40 (50.6)	386 (43.9)
Ex-smoker		55 (39.3)	30 (26.8)	22 (27.9)	253 (28.8)
Never smoked		3 (2.1)	21 (18.8)	9 (11.4)	136 (15.5)
Unknown		11 (7.9)	16 (14.3)	8 (10.1)	105 (11.9)
HCV positive, n (%)		11 (7.9)	5 (4.5)	8 (10.1)	75 (8.5)
HBV positive, n (%)		5 (3.6)	6 (5.4)	6 (7.6)	55 (6.3)
Any use of ARV, n (%)		130 (92.9)	99 (88.4)	78 (98.7)	813 (92.4)
Stage of disease, n (%)	N	76	36	51	Not Collected
Localised		18 (23.7)	6 (16.7)	31 (60.8)	
Disseminated		58 (76.3)	30 (83.3)	20 (39.2)	

### Incidence over time

**Figure 1:**
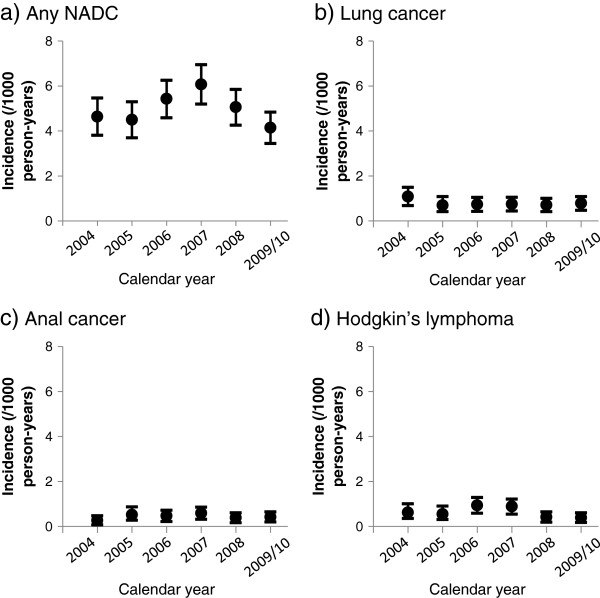
**Crude incidence rate (/1000 PY) of NADC, with 95% confidence intervals, stratified by calendar year. a)** any NADC (880 events), **b)** lung cancer (140 events), **c)** anal cancer (79 events), **d**) Hodgkin’s lymphoma (112).

The mean age of participants increased from 41.3 in 2004 to 46.0 years in 2009/10. The incidence of NADC /1000 PY [95% confidence interval] was 4.64 [3.81-5.47], 4.50 [3.70-5.30], 5.43 [4.59-6.26], 6.07 [5.20-6.95], 5.06 [4.26-5.85] and 4.15 [3.45-4.84] in the years 2004, 2005, 2006, 2007, 2008 and 2009/2010 respectively (Figure 1, p = 0.01, Poisson regression). Age-adjusted incidence rates for these 6 years were 4.64, 4.30, 5.06, 5.42, 4.54 and 3.50/1000 PY respectively. Thus, the incidence of a first NADC did not vary substantially over the study period after age adjustment (p = 0.91, Poisson regression). NADC incidence rates (/1000 PY) were higher in men than in women (5.47 [95% CI 5.07-5.88] vs. 3.63 [3.09-4.17]) and increased with age attained (0.69 [0.33-1.27], 1.70 [1.34-2.06], 4.71 [4.21-5.22], 8.56 [7.50-9.61] and 15.27 [13.16-17.38] in those aged <30, 30–39, 40–49, 50–59 and ≥60 years respectively). Incidence rates were lower in those infected via heterosexual sex (4.15 [3.64-4.66]) than in men-who-have-sex-with-men (MSM) (5.23 [4.72-5.73]), IDU (5.93 [4.99-6.87]) and those of other or unknown mode of HIV acquisition (5.61 [4.23-7.00]).

When considering the three most frequently occurring NADC separately, there continued to be no trend for an increasing or decreasing incidence over time (Figure 1), either before or after adjusting for age. Each of the three most frequently occurring NADC was more common in men than in women and increased in frequency with age (data not shown).

### All-cause mortality following NADC diagnosis

**Figure 2:**
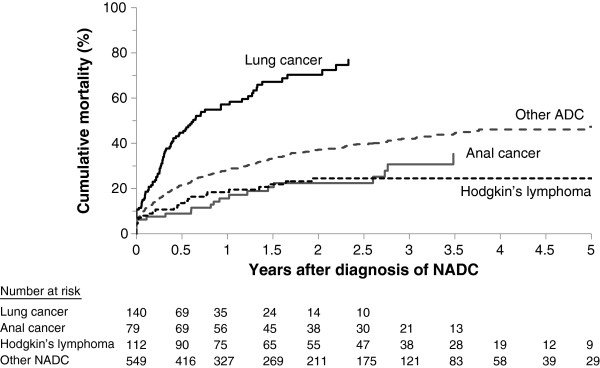
Cumulative mortality following diagnosis of NADC, stratified by type of NADC.

Of the 880 individuals with a NADC, 327 (37.2%) had died of any cause by 1st February 2010. Kaplan-Meier cumulative mortality estimates at 1, 3 and 5 years after NADC diagnosis were 28.2% [95% CI 25.1-31.2], 42.0% [38.2-45.8] and 47.3% [42.4-52.2], respectively, with a median survival time of 5.5 years (Figure 2). Cumulative mortality estimates were particularly high in the 140 individuals diagnosed with lung cancer (57.2% [48.4-65.9] and 77.0% [67.3-86.8] at 1 and 2.5 years, respectively). The 79 individuals diagnosed with anal cancer had cumulative mortality estimates of 15.6% [7.5-23.8] and 30.7% [18.2-43.2] at 1 and 3 years, respectively, whereas the 112 individuals diagnosed with Hodgkin’s lymphoma had cumulative mortality estimates of 18.4% [11.1-25.8] and 24.5% [16.0-33.0].

In total, 289 of the 327 (88.4%) participants who died, died from their NADC, with 4 (1.2%) dying from an ADC, and 8 (2.5%) dying from a non-malignant cause (information was unknown for the remaining 26 (8.0%) participants). The numbers dying from their NADC for the lung cancer, anal cancer and Hodgkin’s lymphoma patients were 84 (94.4%), 17 (85.0%) and 16 (64.0%), respectively, whereas the numbers dying from a non-malignant cause were 1 (1.1%), 2 (10.0%) and 1 (4.0%), respectively. One person (4.0%) with Hodgkin’s lymphoma died from an ADC. Information on the cause of death was unknown for 4 (4.5%), 1 (5.0%) and 7 (28.0%) of the three groups, respectively.

### Predictors of mortality

In univariate analyses, poorer survival after NADC diagnosis was associated with the type of NADC (with those with lung cancer having a much higher mortality rate), male gender, IDU mode of infection, non-white/unknown ethnicity, smoking status, co-infection with HCV or HBV and older age at diagnosis, whereas later year of diagnosis, a higher nadir CD4 count and higher CD4 count at NADC diagnosis were associated with improved survival. No significant association was seen with the HIV RNA level at NADC diagnosis, although the power to detect a significant association was low as most patients were virally suppressed at the time. In adjusted analyses, most of these factors remained associated with mortality (left-hand side, Table 4); the exceptions to this were age, HCV status and the nadir CD4 count, which were no longer associated with mortality after controlling for the patient’s CD4 count at diagnosis and other covariates. There was some evidence that the effect of calendar year strengthened as the time from diagnosis increased, consistent with a reduction in the risk of both cancer-related and non-cancer-related mortality in more recent years. Of interest, neither a previous NADC nor a previous ADC was strongly associated with mortality in univariate analyses.

**Table 4:** Results from multivariable Cox proportional hazards regression models to identify independent associations between factors at the time of diagnosis of NADC and subsequent all-cause mortality and cancer-specific mortality for any NADC, and lung cancer, anal cancer and Hodgkin’s lymphoma separately

		**All-cause mortality**		**Cancer-specific mortality**	
		**RH [95% CI]**	**p-value**	**RH [95% CI]**	**p-value**
***Any NADC***					
Gender	Male	1.69 [1.21, 2.36]	0.002	1.59 [1.11, 2.26]	0.01
	Female	Ref.	-	Ref.	-
Mode of infection	Heterosexual	Ref.	-	Ref.	-
	MSM	0.84 [0.63, 1.11]	0.22	0.84 [0.62, 1.13]	0.24
	IDU	1.35 [0.98, 1.87]	0.07	1.23 [0.87, 1.74]	0.24
	Other/unknown	0.78 [0.48, 1.26]	0.30	0.72 [0.42, 1.22]	0.22
Ethnic group	White	Ref.	-	Ref.	-
	Non-white/other/not collected	1.25 [0.99, 1.56]	0.06	1.18 [0.93, 1.49]	0.19
Smoking status	Current smoker	1.33 [0.88, 1.99]	0.17	1.43 [0.92, 2.23]	0.11
	Ex-smoker	1.79 [1.18, 2.70]	0.006	1.88 [1.20, 2.94]	0.006
	Non-smoker	Ref.	-	Ref.	-
	Unknown	1.60 [0.99, 2.59]	0.05	1.69 [1.01, 2.83]	0.05
HBV status	Positive	1.42 [0.96, 2.12]	0.08	1.38 [0.90, 2.12]	0.14
	Negative	Ref.		Ref.	
Year of diagnosis	/later year	0.93 [0.86, 0.99]	0.04	0.91 [0.84, 0.98]	0.01
CD4 count at diagnosis	/50 cells/mm^3^ higher	0.95 [0.93, 0.98]	0.0001	0.96 [0.94, 0.98]	0.0007
Type of NADC	Lung cancer	2.28 [1.75, 2.98]	0.0001	2.43 [1.84, 3.21]	0.0001
	Anal cancer	0.58 [0.36, 0.92]	0.02	0.55 [0.33, 0.92]	0.02
	Hodgkin’s lymphoma	0.49 [0.32, 0.75]	0.001	0.36 [0.21, 0.60]	0.001
	Other	Ref.	-	Ref.	-
***Lung cancer***					
HCV status	Positive	1.88 [0.97, 3.66]	0.06	2.02 [1.04, 3.95]	0.04
	Negative	Ref.	-	Ref.	-
Stage of disease	Disseminated	5.22 [1.85, 14.76]	0.002	4.84 [1.71, 13.74]	0.003
	Localised	Ref.	-	Ref.	-
	Unknown	5.95 [2.12, 16.74]	0.0007	5.69 [2.02, 16.06]	0.001
***Anal cancer***					
HCV status	Positive	4.97 [1.55, 15.89]	0.007	4.16 [1.18, 14.67]	0.03
	Negative	Ref.	-	Ref.	-
Previous NADC		12.00 [2.98, 48.32]	0.0005	10.17 [1.96, 52.73]	0.006
Stage of disease	Disseminated	2.49 [0.73, 8.54]	0.15	2.29 [0.65, 8.10]	0.20
	Localised	Ref.	-	Ref.	-
	Unknown	3.24 [0.94, 11.18]	0.06	2.43 [0.66, 8.95]	0.18
***Hodgkin’s lymphoma***					
HBV status	Positive	4.15 [1.16, 14.90]	0.03	3.32 [0.68, 16.21]	0.14
	Negative	Ref.	-	Ref.	-
CD4 count at diagnosis	/50 cells/mm^3 ^higher	0.86 [0.74, 1.01]	0.06	0.80 [0.64, 0.99]	0.04
Haemoglobin at diagnosis	/g/L higher	0.72 [0.55, 0.94]	0.02	0.82 [0.59, 1.14]	0.24

Analyses of risk factors for mortality after diagnosis of specific NADC were limited due to the relatively small number of patients experiencing these events. However, in multivariable analyses, HCV co-infection and disseminated cancer at time of diagnosis were predictors of mortality among patients with lung cancer (Table 4). Risk factors for poorer survival after anal cancer were a previous NADC, HCV co-infection and disseminated cancer at time of diagnosis. Finally, HBV positivity, lower haemoglobin level at NADC diagnosis and lower CD4 cell count were associated with mortality after Hodgkin’s lymphoma. Of note, no association was seen in univariate analyses between the latest CD4 count and mortality from either lung (relative hazard per 50 cells/mm^3^ higher: 0.99 [95% confidence interval 0.95-1.03], p = 0.70) or anal (1.02 [0.93-1.12], p = 0.70) cancer.

In sensitivity analyses with an endpoint of cancer-specific mortality (right-hand side, Table 4), we found similar associations to those seen for all-cause mortality for gender, smoking status, year of diagnosis, HBV status, CD4 count at diagnosis and type of cancer. However, the associations previously seen with IDU mode of HIV acquisition and non-white/other/unknown ethnicity were reduced and were non-significant. When our analyses were restricted to individuals diagnosed with lung cancer, anal cancer or HL, associations were again broadly similar to those seen for all-cause mortality, although due to the reduced power of these analyses, confidence intervals for any effects were generally wider.

## Discussion

Using an international cohort with centrally validated endpoints, we describe 880 NADC diagnosed from 2004–2010. The overall incidence of NADC in this cohort of HIV-positive persons was 4.98/1000 PY (498/100,000 PY) with no evidence of an increasing or decreasing incidence over the study period. Survival after NADC diagnosis was poor (median 5.5 years), with over a third of patients dying; survival varied substantially depending on the type of NADC.

Published estimates of the incidence of NADC vary widely. For example, whilst our reported incidence is similar to that in many studies [5,7,20,23], it is substantially lower than reported in one study [8] and higher than that in three others [6,24,25]. Of note, incidence estimates from some other studies e.g. [2,3,9] should be interpreted with caution due to the restriction of the population to those with AIDS, a group potentially at higher risk of cancer due to their advanced stage of immunodeficiency [23]. A direct comparison of the changes in NADC incidence over time in these studies is also difficult, as many of the earlier studies compared changes in NADC incidence in the pre-and post-cART eras. Both the ageing and prolonged exposure to immune suppression that have occurred in the post-cART era may have resulted in an increased cancer incidence. However, with this caveat, findings regarding a change in NADC incidence have been inconsistent, with some studies reporting an increase in incidence over time [3,4,6,8,9] but others either not seeing an increase [24] or reporting an increase for specific NADC only [2,5]. Of interest, whilst one large cohort of HIV-positive individuals reported an increase in the incidence of NADC from 1983–2001, the increase did not continue over the period 2002–2007 [6], consistent with our finding of no increase over the period 2004–2010.

Our findings of a stable incidence over time for anal cancers are consistent with some studies [7,10,11]. In contrast, several other studies have reported an increase in incidence [3-5,9,26], which may also be apparent in the non-HIV population [27]. As noted earlier, the majority of these studies compared incidence rates to the pre-cART era, which may explain the different conclusions. Furthermore, not all studies have taken account of the ageing of the underlying population, which may be expected to lead to an increase in the overall cancer rate. However, it is also possible that differential ascertainment of anal cancers between studies that are registry-based (which may, in some countries, include collection of data on pre-cancers) and studies that require confirmation by histology, may contribute to the inconsistent findings. Finally, trends may also be affected by any changes to local and national anal cancer screening policies: reports from the participating D:A:D cohorts suggest that screening in HIV clinics has not generally been performed frequently or homogeneously across participating countries. Hence, we do not believe that this is likely to be a major source of bias.

Poorer survival after NADC diagnosis was associated with male gender, IDU mode of infection, non-white ethnicity, smoking status, coinfection with hepatitis B virus and earlier year of NADC diagnosis. Similar associations have been reported previously [8,13,18,19]. The improved survival in recent years may reflect an increased use of cancer treatment in HIV-positive patients, as well as the availability of improved ART regimens leading to greater CD4 count increases, and a reduction in cancer risk behaviour (e.g. increased smoking cessation). Of note, an individual’s CD4 count at NADC diagnosis remains a strong predictor of subsequent mortality for all NADC combined (as well as for Hodgkin’s lymphoma), emphasising the need for continued use of cART, even in those with NADC. It is interesting that HCV and HBV co-infection were associated with a higher mortality following any cancer (HBV), lung cancer (HCV), anal cancer (HCV) and Hodgkin’s lymphoma (HBV); whilst co-infection itself may lead to higher mortality, viral co-infection may also be a marker for other lifestyle factors that may by themselves place the individual at a higher risk of mortality. Whilst older age was significantly associated with survival following NADC diagnosis in univariate analyses, it did not remain significantly associated with survival in adjusted analyses; this may reflect the relatively restricted age of the underlying cohort population, and of cohort participants at the time of NADC diagnosis.

Although there has been some suggestion that the incidence of NADC is higher in patients with AIDS [28], we did not find strong associations between mortality and either a previous NADC or a previous ADC, with the exception of anal cancer. We observed several important findings with regard to mortality and risk factors for some specific NADC. Unsurprisingly, lung cancers had the highest mortality rate, with 94% of those who died dying from the lung cancer itself. This is a similar rate to that reported from a study of lung cancer in injection drug users [29]. It is noteworthy that in our study there was no association between the CD4 count at lung cancer diagnosis and subsequent mortality, consistent with no effect of HIV on survival after this diagnosis. However, the CD4 count was associated with mortality following a diagnosis of Hodgkin’s lymphoma and any NADC overall. As most of the deaths that did occur were cancer-related, these findings may highlight a possible role of immune suppression in the development, and subsequent control, of cancers. Subsequent analyses of this cohort will investigate associations with immune suppression in more detail. However, it is important to note that other explanations for an association between survival and the CD4 count cannot be ruled out. For example, the finding may reflect bias that may be introduced if patients with lower CD4 counts were not offered, or could not tolerate anti-cancer treatments (we have limited data on use of chemo- and/or radio-therapy, or on surgical treatments for cancer).

Our mortality rates among patients with Hodgkin’s lymphoma are similar to those previously reported in the setting of HIV perhaps reflecting our contemporary HIV population where the majority are well treated on cART and with access to appropriate cancer care. One study reported the impact of cART [30] on mortality, another the impact of chemotherapy on advanced disease [31]. Somewhat larger discrepancies have been reported in relation to survival after an anal cancer diagnosis – these could be explained by the inclusion of pre-cancers or *in situ* cancers in some of the registry-based studies (which may be expected to show better survival outcomes) and a hypothetically increased use of different treatment modalities in recent years. Our mortality rate at two years (30%) is similar to that reported from recent prospective studies in the UK [32] and the US [19], whereas our reported five-year mortality rate (35%) is somewhat lower than that from a smaller retrospective study exploring the impact of chemotherapy and surgery in HIV-positive and HIV-negative individuals (61%) [33]. As this study was performed over an earlier time-period to our own and included patients with non-metastatic invasive anal cancer, the results may not be directly comparable.

We repeated our analyses of predictors of all-cause mortality for the outcome cancer-specific mortality. As such a large proportion of the deaths were due to cancer, we did not expect the results to differ greatly, and this was generally the case. Interestingly, when considering cancer-related mortality following all NADC combined, the associations previously seen with IDU mode of HIV acquisition and non-white/other/unknown ethnicity were weakened. This presumably reflecting a stronger association of these factors with other, non-cancer, causes of death, although lack of power cannot be ruled out as a possible explanation. When our analyses were restricted to individuals diagnosed with lung cancer, anal cancer or HL, associations were similar to those reported for all-cause mortality. Of note, whilst these analyses provide some interesting findings, they address a somewhat different question. From a clinical perspective, it is impossible to know in advance whether the patient will die of his/her underlying cancer or of a different cause, and hence our primary analyses maybe of more relevance to the treating physician.

Our study has several limitations. Unfortunately, information on dissemination or stage of disease at diagnosis, important risk factors for survival, was unavailable for most patients. Furthermore, we do not systematically collect information on socioeconomic status or alcohol consumption as part of the D:A:D Study, both of which are likely to be associated with survival outcomes. We do suffer from some missing and incomplete data, particularly regarding smoking and HBV/HCV coinfection status. As our analysis has focused on the first NADC during prospective D:A:D follow-up for each individual, we may have under-estimated the incidence of some NADC, particularly those that may more commonly occur as secondary cancers. However, only 46 patients with a NADC in the present analysis developed a subsequent cancer, suggesting that the impact of any under-estimation will be small. Finally, whilst we recognize that individual NADC are very different, in terms of their pathogenesis, natural history and therapeutic possibilities, the low incidence of most specific types of NADC meant that we were unable to consider these separately (other than the three most commonly occurring NADC). Even for the three most commonly occurring NADC, our multivariable models to identify predictors of survival were restricted by low power. Thus, our analyses were only able to identify the strongest predictors of survival and we cannot rule out the possibility that other factors may also be associated with survival outcomes. Further follow-up of this study may permit more detailed analyses of specific NADC in the future.

## Conclusions

In conclusion, the incidence of NADC from 2004–10 has been stable in this large observational cohort with an overall incidence of NADC of 4.98/1000 PY. The prognosis after diagnosis of NADC, in particular lung cancer and disseminated cancer, is poor but has improved somewhat over time. A higher CD4 count at diagnosis was associated with improved survival for some cancers, and when considering NADC jointly. Earlier diagnosis of both HIV and NADC, and research into better management of NADC is warranted.

## Appendix

D:A:D participating cohorts and Steering Committee (names marked with *)

**Members of the D:A:D SC from the Oversight Committee:** N. Shortman*, D. Butcher*, R. Rode*, X. Franquet *, W. Powderly*

**D:A:D Central Coordination:** L. Ryom, C.A. Sabin*, D. Kamara, C. Smith, A. Phillips*, A. Mocroft, J. Tverland, J. Nielsen, J.D. Lundgren (chair)

**D:A:D data managers:** R. Salbøl Brandt (coordinator), M. Rickenbach, I. Fanti, E. Krum, M. Hillebregt, S. Geffard, A. Sundström, M. Delforge, E. Fontas, F. Torres, H. McManus, S. Wright, J. Kjær.

**Endpoint verification Group**: A. Sjøl (CVD primary endpoint), P. Meidahl (oncology), J. Helweg-Larsen (hematology), J. Schmidt Iversen (nephrology).

The members of the 11 Cohorts are as follows:

**ATHENA** (AIDS Therapy Evaluation Project Netherlands):

**Central coordination:** F. de Wolf, S. Zaheri, M Hillebregt L. Gras;

**Participating physicians** (¤Site coordinating physicians): **Academisch Medisch Centrum bij de Universiteit van Amsterdam, Amsterdam**: J.M. Prins¤, T.W. Kuijpers, H.J. Scherpbier, K. Boer, J.T.M. van der Meer, F.W.M.N. Wit, M.H. Godfried, P. Reiss*, T. van der Poll, F.J.B. Nellen, J.M.A. Lange, S.E. Geerlings, M. van Vugt, S.M.E. Vrouenraets, D. Pajkrt, M. van der Valk. **Academisch Ziekenhuis Maastricht, Maastricht**: G. Schreij¤, S. Lowe, A. Oude Lashof. **Catharina-ziekenhuis, Eindhoven**: M.J.H. Pronk¤, B. Bravenboer. **Erasmus Medisch Centrum, Rotterdam**: M.E. van der Ende¤, T.E.M.S. de Vries-Sluijs, C.A.M. Schurink, M. van der Feltz, J.L. Nouwen, L.B.S. Gelinck, A. Verbon, B.J.A. Rijnders, L. Slobbe. **Erasmus Medisch Centrum–Sophia, Rotterdam**: N.G. Hartwig, G.J.A. Driessen. **Flevoziekenhuis, Almere**: J. Branger¤. **HagaZiekenhuis, Den Haag**: R.H. Kauffmann¤, E.F. Schippers. **Isala Klinieken, Zwolle**: P.H.P. Groeneveld¤, M.A. Alleman, J.W. Bouwhuis. **Kennemer Gasthuis**: R.W. ten Kate¤, R. Soetekouw. **Leids Universitair Medisch Centrum, Leiden**: F.P. Kroon¤, P.J. van den Broek, J.T. van Dissel, S.M. Arend, C. van Nieuwkoop, M.G.J. de Boer, H. Jolink. **Maasstadziekenhuis, Rotterdam**: J.G. den Hollander¤, K. Pogany. **Medisch Centrum Alkmaar, Alkmaar**: G. van Twillert¤, W. Kortmann. **Medisch Centrum Haaglanden, Den Haag**: R. Vriesendorp, ¤ E.M.S. Leyten. **Medisch Spectrum Twente, Enschede**: C.H.H. ten Nape¤l, G.J. Kootstra. **Onze Lieve Vrouwe Gasthuis, Amsterdam**: K. Brinkman¤, W.L. Blok, P.H.J. Frissen, W.E.M. Schouten, G.E.L. van den Berk. **Sint Elisabeth Ziekenhuis, Tilburg**: J.R. Juttmann¤, M.E.E. van Kasteren, A.E. Brouwer. **Sint Lucas Andreas Ziekenhuis, Amsterdam**: J. Veenstra ¤,K.D. Lettinga. **Slotervaartziekenhuis, Amsterdam**: J.W. Mulder¤, E.C.M. van Gorp, P.M. Smit, S. Weijer. **Stichting Medisch Centrum Jan van Goyen, Amsterdam**: A. van Eeden*, D.W.M. Verhagen¤. **Universitair Medisch Centrum Groningen, Groningen**: H.G. Sprenger¤, R. Doedens, E.H. Scholvinck, S. van Assen, C.J. Stek. **Universitair Medisch Centrum Sint Radboud, Nijmegen**: P.P. Koopmans¤, R. de Groot, M. Keuter, A.J.A.M. van der Ven, H.J.M. ter Hofstede, M. van der Flier, A.M. Brouwer, A.S.M. Dofferhoff. **Universitair Medisch Centrum Utrecht, Utrecht**: A.I.M. Hoepelman¤, T. Mudrikova, M.M.E. Schneider, C.A.J.J. Jaspers, P.M. Ellerbroek, E.J.G. Peters, L.J. Maarschalk-Ellerbroek, J.J. Oosterheert, J.E. Arends, M.W.M. Wassenberg, J.C.H. van der Hilst. **Vrije Universiteit Amsterdam, Amsterdam**: S.A. Danner¤, M.A. van Agtmael, J. de Vocht, R.M. Perenboom, F.A.P. Claessen, W.F.W. Bierman, E.V. de Jong, E.A. bij de Vaate. **Wilhelmina Kinderziekenhuis, Utrecht**: S.P.M. Geelen, T.F.W. Wolfs. **Ziekenhuis Rijnstate, Arnhem**: C. Richter, ¤ J.P. van der Berg, E.H. Gisolf. **Ziekenhuis Walcheren, Vlissingen**: M. van den Berge ¤, A. Stegeman. **Medisch Centrum Leeuwarden, Leeuwarden**: D.P.F. van Houte¤, M.B. Polée, M.G.A. van Vonderen. **Sint Elisabeth Hospitaal, Willemstad - Curaçao**: C. Winkel, A.J. Duits.

**ANRS CO3 Aquitaine Cohort (**France)

**Composition of the Groupe d’Epidémiologie Clinique du Sida en Aquitaine (GECSA)**:

**Coordination:** F. Dabis. **Scientific committee**: F. Bonnet, F. Dabis, M. Dupon, G. Chêne, H. Fleury, D. Lacoste, D. Malvy, P. Mercié, I. Pellegrin, P. Morlat, D. Neau, JL. Pellegrin, R. Thiébaut, K. Titier. **Epidemiology and Methodology**: M. Bruyand, G. Chêne, F. Dabis, S. Lawson-Ayayi, R. Thiébaut, L. Wittkop. **Infectious Diseases and Internal Medicine**: F. Bonnal, F. Bonnet, N. Bernard, L. Caunègre, C. Cazanave, J. Ceccaldi, D. Chambon, I. Chossat, K. Courtaud, FA. Dauchy, S. De Witte, M. Dupon, A. Dupont, P. Duffau, H. Dutronc, S. Farbos, V. Gaboriau, MC. Gemain, Y. Gerard, C. Greib, M. Hessamfar, D. Lacoste, P. Lataste, S. Lafarie-Castet, E. Lazaro, M. Longy-Boursier, D. Malvy, JP. Meraud, P. Mercié, E. Monlun, P. Morlat, D. Neau, A. Ochoa, JL. Pellegrin, T. Pistone, JM. Ragnaud, MC. Receveur, J. Roger-Schmeltz, S. Tchamgoué, P. Thibaut, MA. Vandenhende, JF. Viallard. **Immunology**: JF. Moreau, I. Pellegrin. Virology: H. Fleury, ME. Lafon, B. Masquelier, P. Trimoulet. **Pharmacology**: D. Breilh, K. Titier. **Drug monitoring**: F. Haramburu, G. Miremont-Salamé. **Data collection and processing**: MJ. Blaizeau, M. Decoin, J. Delaune, S. Delveaux, C. D’Ivernois, C. Hanappier, O. Leleux, B. Uwamaliya-Nziyumvira, X. Sicard. **Computing and Statistical analysis**: S. Geffard, J. Leray, G. Palmer, D. Touchard.

**AHOD** (Australian HIV Observational Database, Australia):

**Central coordination**: M. Law *, K. Petoumenos, H. McManus, S. Wright, C. Bendall (Sydney, New South Wales);

**Participating physicians** (city, state): R. Moore, S. Edwards, J. Hoy, K. Watson, N. Roth, J. Nicholson (Melbourne, Victoria); M Bloch, T. Franic, D. Baker, R. Vale, A. Carr, D. Cooper (Sydney, New South Wales); J. Chuah, M. Ngieng (Gold Coast, Queensland), D. Nolan, J. Skett (Perth, Western Australia).

**BASS** (Spain):

**Central coordination**: G. Calvo, F. Torres, S. Mateu (Barcelona);

**Participating physicians** (city): P. Domingo, M.A. Sambeat, J. Gatell, E. Del Cacho, J. Cadafalch, M. Fuster (Barcelona); C. Codina, G. Sirera, A. Vaqué (Badalona).

**The Brussels St Pierre Cohort** (Belgium): Coordination: S. De Wit*, N. Clumeck, M. Delforge, C. Necsoi. Participating physicians: N. Clumeck, S. De Wit*, AF Gennotte, M. Gerard, K. Kabeya, D. Konopnicki, A. Libois, C. Martin, M.C. Payen, P. Semaille, Y. Van Laethem.

**CPCRA** (USA):

**Central coordination**: J. Neaton, G. Bartsch, W.M. El-Sadr*, E. Krum, G. Thompson, D. Wentworth; **Participating physicians** (city, state): R. Luskin-Hawk (Chicago, Illinois); E. Telzak (Bronx, New York); W.M. El-Sadr (Harlem, New York); D.I. Abrams (San Francisco, California); D. Cohn (Denver, Colorado); N. Markowitz (Detroit, Michigan); R. Arduino (Houston, Texas); D. Mushatt (New Orleans, Louisiana); G. Friedland (New Haven, Connecticut); G. Perez (Newark, New Jersey); E. Tedaldi (Philadelphia, Pennsylvania); E. Fisher (Richmond, Virginia); F. Gordin (Washington, DC); L.R. Crane (Detroit, Michigan); J. Sampson (Portland, Oregon); J. Baxter (Camden, New Jersey).

**EuroSIDA** (multinational**) Coordinating Centre**: J. Lundgren#, O. Kirk*, A. Mocroft, A. Cozzi-Lepri, D. Grint, D. Podlekareva, J. Kjær, L. Peters, J. Reekie, J. Kowalska, J. Tverland, A.H. Fischer, J. Nielsen **Participating countries and physicians Argentina**: (M. Losso), C. Elias, Hospital JM Ramos Mejia, Buenos Aires.

**Austria**: (N. Vetter), Pulmologisches Zentrum der Stadt Wien, Vienna; R. Zangerle, Medical University Innsbruck, Innsbruck.

**Belarus**: (I. Karpov), A. Vassilenko, Belarus State Medical University, Minsk; V.M. Mitsura, Gomel State Medical University, Gomel; O. Suetnov, Regional AIDS Centre, Svetlogorsk.

**Belgium**: (N. Clumeck), S. De Wit*, M Delforge, Saint-Pierre Hospital, Brussels; R. Colebunders, Institute of Tropical Medicine, Antwerp; L. Vandekerckhove, University Ziekenhuis Gent, Gent.

**Bosnia-Herzegovina**: (V. Hadziosmanovic), Klinicki Centar Univerziteta Sarajevo, Sarajevo.

**Bulgaria**: (K. Kostov), Infectious Diseases Hospital, Sofia.

**Croatia**: (J. Begovac), University Hospital of Infectious Diseases, Zagreb.

**Czech Republic**: (L. Machala), D. Jilich, Faculty Hospital Bulovka, Prague; D. Sedlacek, Charles University Hospital, Plzen.

**Denmark**: (J. Nielsen), G. Kronborg,T. Benfield, M. Larsen, Hvidovre Hospital, Copenhagen; J. Gerstoft, T. Katzenstein, A-B.E. Hansen, P. Skinhøj, Rigshospitalet, Copenhagen; C. Pedersen, Odense University Hospital, Odense; L. Ostergaard, Skejby Hospital, Aarhus.

**Estonia**: (K. Zilmer), West-Tallinn Central Hospital, Tallinn; J. Smidt, Nakkusosakond Siseklinik, Kohtla-Järve.

**Finland**: (M. Ristola), Helsinki University Central Hospital, Helsinki.

**France**: (C. Katlama), Hôpital de la Pitié-Salpétière, Paris; J-P. Viard, Hôpital Necker-Enfants Malades, Paris; P-M. Girard, Hospital Saint-Antoine, Paris; J.M. Livrozet, Hôpital Edouard Herriot, Lyon; P. Vanhems, University Claude Bernard, Lyon; C. Pradier, Hôpital de l’Archet, Nice; F. Dabis*, D. Neau, Unité INSERM, Bordeaux.

**Germany**: (J. Rockstroh), Universitäts Klinik Bonn; R. Schmidt, Medizinische Hochschule Hannover; J. van Lunzen, O. Degen, University Medical Center Hamburg-Eppendorf, Infectious Diseases Unit, Hamburg; H.J. Stellbrink, IPM Study Center, Hamburg; S. Staszewski, JW Goethe University Hospital, Frankfurt; J. Bogner, Medizinische Poliklinik, Munich; G. Fätkenheuer, Universität Köln, Cologne.

**Greece**: (J. Kosmidis), P. Gargalianos, G. Xylomenos, J. Perdios, Athens General Hospital; G. Panos, A. Filandras, E. Karabatsaki, 1st IKA Hospital; H. Sambatakou, Ippokration Genereal Hospital, Athens.

**Hungary**: (D. Banhegyi), Szent Lásló Hospital, Budapest.

**Ireland**: (F. Mulcahy), St. James’s Hospital, Dublin.

**Israel**: (I. Yust), D. Turner, M. Burke, Ichilov Hospital, Tel Aviv; S. Pollack, G. Hassoun, Rambam Medical Center, Haifa; S. Maayan, Hadassah University Hospital, Jerusalem. **Italy**: (S. Vella), Istituto Superiore di Sanità, Rome; R. Esposito, I. Mazeu, C. Mussini, Università Modena, Modena; C. Arici, Ospedale Riuniti, Bergamo; R. Pristera, Ospedale Generale Regionale, Bolzano; F. Mazzotta, A. Gabbuti, Ospedale S Maria Annunziata, Firenze; V. Vullo, M. Lichtner, University di Roma la Sapienza, Rome; A. Chirianni, E. Montesarchio, M. Gargiulo, Presidio Ospedaliero AD Cotugno, Monaldi Hospital, Napoli; G. Antonucci, A. Testa, P. Narciso, C. Vlassi, M. Zaccarelli, Istituto Nazionale Malattie Infettive Lazzaro Spallanzani, Rome; A. Lazzarin, A. Castagna, N. Gianotti, Ospedale San Raffaele, Milan; M. Galli, A. Ridolfo, Osp. L. Sacco, Milan; A. d’Arminio Monforte*, Istituto Di Clinica Malattie Infettive e Tropicale, Milan.

**Latvia**: (B. Rozentale), I. Zeltina, Infectology Centre of Latvia, Riga.

**Lithuania**: (S. Chaplinskas), Lithuanian AIDS Centre, Vilnius.

**Luxembourg**: (R. Hemmer), T. Staub, Centre Hospitalier, Luxembourg.

**Netherlands**: (P. Reiss*), Academisch Medisch Centrum bij de Universiteit van Amsterdam, Amsterdam.**Norway**: (V. Ormaasen), A. Maeland, J. Bruun, Ullevål Hospital, Oslo.

**Poland**: (B. Knysz), J. Gasiorowski, Medical University, Wroclaw; A. Horban, E. Bakowska, Centrum Diagnostyki i Terapii AIDS, Warsaw; A. Grzeszczuk, R. Flisiak, Medical University, Bialystok; A. Boron-Kaczmarska, M. Pynka, M. Parczewski, Medical Univesity, Szczecin; M. Beniowski, E. Mularska, Osrodek Diagnostyki i Terapii AIDS, Chorzow; H. Trocha, Medical University, Gdansk; E. Jablonowska, E. Malolepsza, K. Wojcik, Wojewodzki Szpital Specjalistyczny, Lodz.

**Portugal**: (F. Antunes), M. Doroana, L. Caldeira, Hospital Santa Maria, Lisbon; K Mansinho, Hospital de Egas Moniz, Lisbon; F. Maltez, Hospital Curry Cabral, Lisbon.

**Romania**: (D. Duiculescu), Spitalul de Boli Infectioase si Tropicale: Dr. Victor Babes, Bucarest.

**Russia**: (A. Rakhmanova), Medical Academy Botkin Hospital, St Petersburg; N. Zakharova, St Petersburg AIDS Centre, St Peterburg; S. Buzunova, Novgorod Centre for AIDS, Novgorod.

**Serbia**: (D. Jevtovic), The Institute for Infectious and Tropical Diseases, Belgrade.

**Slovakia**: (M. Mokráš), D. Staneková, Dérer Hospital, Bratislava.

**Slovenia**: (J. Tomazic), University Clinical Centre Ljubljana, Ljubljana.

**Spain**: (J. González-Lahoz), V. Soriano, P. Labarga, J. Medrano, Hospital Carlos III, Madrid; S. Moreno, J.M. Rodriguez, Hospital Ramon y Cajal, Madrid; B. Clotet, A. Jou, R. Paredes, C. Tural, J. Puig, I. Bravo, Hospital Germans Trias i Pujol, Badalona; J.M. Gatell, J.M. Miró, Hospital Clinic i Provincial, Barcelona; P. Domingo, M. Gutierrez, G. Mateo, M.A. Sambeat, Hospital Sant Pau, Barcelona.**Sweden**: (A. Karlsson), Venhaelsan-Sodersjukhuset, Stockholm; L. Flamholc, Malmö University Hospital, Malmö. **Switzerland**: (B. Ledergerber), R. Weber*, University Hospital, Zürich; P. Francioli, M. Cavassini, Centre Hospitalier Universitaire Vaudois, Lausanne; B. Hirschel, E. Boffi, Hospital Cantonal Universitaire de Geneve, Geneve; H. Furrer, Inselspital Bern, Bern; M. Battegay, L. Elzi, University Hospital Basel. **Ukraine**: (E. Kravchenko), N. Chentsova, Kiev Centre for AIDS, Kiev; V. Frolov, G. Kutsyna, Luhansk State Medical University; Luhansk; S. Servitskiy, Odessa Region AIDS Center, Odessa; M. Krasnov, Kharkov State Medical University, Kharkov. **United Kingdom**: (S. Barton), St. Stephen’s Clinic, Chelsea and Westminster Hospital, London; A.M. Johnson, D. Mercey, University College London, London (University College Campus); A. Phillips, M.A. Johnson, A. Mocroft, Royal Free Hospital and University College London, London (Royal Free Campus); M. Murphy, Medical College of Saint Bartholomew’s Hospital, London; J. Weber, G. Scullard, Imperial College School of Medicine at St. Mary’s, London; M. Fisher, Royal Sussex County Hospital, Brighton; C. Leen, Western General Hospital, Edinburgh.

**HivBivus** (Sweden):

**Central coordination**: L. Morfeldt, G. Thulin, A. Sundström.

**Participating physicians (city)**: B. Åkerlund (Huddinge); K. Koppel, A. Karlsson (Stockholm); L. Flamholc, C. Håkangård (Malmö).

**The IcoNA Foundation Study (Italy)**:

**GOVERNING BODY**: M. Moroni (Chair), G. Angarano, A. Antinori, F. Castelli, R. Cauda, A. d’Arminio Monforte, G. Di Perri, M. Galli, R. Iardino, G. Ippolito, A. Lazzarin, C.F. Perno, O. Armignacco, P.L. Viale, F. Von Schlosser.

**SCIENTIFIC SECRETARY**: A. d’Arminio Monforte

**STEERING COMMITTEE**: A. Ammassari, M. Andreoni, A. Antinori, C. Balotta, P. Bonfanti, S. Bonora, M. Borderi, M.R. Capobianchi, A. Castagna, F. Ceccherini-Silberstein, P. Cinque, A. Cozzi-Lepri, A. d’Arminio Monforte, A. De Luca, M. Gargiulo, C. Gervasoni, E. Girardi, A. Gori, G. Guaraldi, M. Lichtner, S. Lo Caputo, G. Madeddu, F. Maggiolo, G. Marchetti, S. Marcotullio, L. Monno, R. Murri, C. Mussini, M. Puoti, C. Torti.

**STATISTICAL AND MONITORING TEAM**: A. Cozzi-Lepri, P. Cicconi, I. Fanti, T. Formenti, L. Galli, P. Lorenzini.

**PARTICIPATING PHYSICIANS AND CENTERS**:** Italy** A. Giacometti, A Costantini, A. Riva (Ancona); G. Angarano, L. Monno, C. Carrisa, (Bari); F. Maggiolo, G. Lazzari (Bergamo); P.L. Viale, M. Borderi, G. Verucchi (Bologna); F. Castelli, C. Torti, C. Minardi, (Brescia); T. Quirino, C. Abeli (Busto Arsizio); P.E. Manconi, P. Piano (Cagliari); J. Vecchiet, K. Falasca (Chieti); L. Sighinolfi, D. Segala (Ferrara); F. Mazzotta, S. Lo Caputo (Firenze); G. Cassola, G. Viscoli, A. Alessandrini, R. Piscopo, G. Mazzarello (Genova); C. Mastroianni, V. Belvisi (Latina); P. Bonfanti, I. Caramma (Lecco); A. Chiodera, P. Castelli (Macerata); M. Galli, A. Lazzarin, G. Rizzardini, M. Puoti, A. d’Arminio Monforte, A.L. Ridolfo, R. Piolini, A. Castagna, S. Salpietro, A. Galli, A. Bigoloni, V. Spagnuolo, L. Carenzi, P. Zucchi, M.C. Moioli, R. Rossotti, P. Cicconi, T. Formenti (Milano); C. Mussini, L. Bisio (Modena); A. Gori, G. Lapadula (Monza), N. Abrescia, A. Chirianni, M.G. Guida, M. Gargiulo (Napoli); F. Baldelli, B. Belfiori (Perugia); G. Parruti, T. Ursini (Pescara); G. Magnani, M.A. Ursitti (Reggio Emilia); R. Cauda, M. Andreoni, A. Antinori, V. Tozzi, V. Vullo, A. De Luca, A. d’Avino, M. Zaccarelli, L. Gallo, E. Nicastro, R. Acinapura, M. Capozzi, R. Libertone, M. Lichtner, G. Tebano, (Roma); M.S. Mura, G. Madeddu (Sassari); P. Caramello, G. Di Perri, G.C. Orofino, M. Sciandra (Torino); G. Pellizzer, V. Manfrin (Vicenza).

**Nice HIV Cohort** (France):

**Central coordination**: C. Pradier*, E. Fontas, C. Caissotti.

**Participating physicians**: P. Dellamonica, E. Bernard, E. Cua, F. De Salvador-Guillouet, J. Durant, S. Ferrando, V. Mondain-Miton, A. Naqvi, I. Perbost, B. Prouvost-Keller, S. Pillet, P. Pugliese, V. Rahelinirina, P.M. Roger.

**Clinical research assistant**: K. Dollet

**SHCS** (Swiss HIV Cohort Study, Switzerland): J. Barth, M. Battegay, E. Bernasconi, J. Böni, H.C. Bucher, C. Burton-Jeangros, A. Calmy, M. Cavassini, C. Cellerai, R. Dubs, M. Egger, L. Elzi, J. Fehr, M. Flepp, P. Francioli (President of the SHCS), H. Furrer, C.A. Fux, M. Gorgievski, H. Günthard, B. Hasse, H.H. Hirsch, B. Hirschel, I. Hösli, C. Kahlert, L. Kaiser, O. Keiser, C. Kind, T. Klimkait, H. Kovari, B. Ledergerber, G. Martinetti, B. Martinez de Tejada, N. Müller, D. Nadal, G. Pantaleo, A. Rauch, S. Regenass, M. Rickenbach, C. Rudin, P. Schmid, D. Schultze, F. Schöni-Affolter, J. Schüpbach, R. Speck, P. Taffé, A. Telenti, A. Trkola, P. Vernazza, V. von Wyl, R. Weber*, S. Yerly.

## Abbreviations

NADC: Non-AIDS-defining cancers; ADC: AIDS-defining cancers; cART: Combination antiretroviral therapy; CRF: Case report form; HIV: Human immunodeficiency virus; HBV: Hepatitis B virus; HCV: Hepatitis C virus; IQR: Inter-quartile range; PY: Person-years; MSM: Men who have sex with men; IDU: Injection drug users.

## Competing interests

The authors declare that they have no competing interests.

## Authors’ contributions

SWW, CAS and JDL developed the initial study protocol with input from MB, FB, GF, DIA, AG and HF. SWW co-ordinated the study, prepared the datasets for analysis and assisted with endpoint review. CAS performed all statistical analysis and, with SWW, prepared the initial draft of the manuscript. PR, ML, AD’AM, EF, OK, SdW, AP and JDL have provided management input to the D:A:D Study, contributed datasets, provided input to the development of the manuscript and have given final approval for it to be published.

## Authors’ information

For the D:A:D Study Group: Please see Appendix for full Study Group listing.

## Pre-publication history

The pre-publication history for this paper can be accessed here:

http://www.biomedcentral.com/1471-2334/13/471/prepub
